# Water: Its History, Characteristics, Hygienic, and Therapeutic Uses

**Published:** 1862-01

**Authors:** Samuel W. Francis

**Affiliations:** New York


### Selections.

WATER: ITS HISTORY, CHARACTERISTICS, HYGIENIC, AND THERAPEUTIC USES.—By *Samuel W. Francis*, A. M., M. D., *of New York*,—IN NATURAL PHILOSOPHY.—Within the wide range of scientific investigations, there is nothing so pleasing to the contemplative mind, nought that brings as vividly before the imagination, nature's certitudes, as the various branches of Natural Philosophy. With a clear conception of what is æsthetic in its character, and convinced of the capabilities of universal laws, the student of the past reaps vast satisfaction and obtains a priceless treasure by following, conscientiously, the by-paths of a subtle element; or tracing out for future usefulness the powerful workings of a hidden principle.

Many wonders do exist surpassing strange and full of meaning, but, to the inspired reader of what has been done, a beauteous vista in the happy future holds out tempting prizes for the indefatigable. Perseverance and unimpaired zeal surmount all obstacles. Man may pronounce, in public, what appears most futile for a healthy mind to comprehend; but innate monitors predict the birth of what some century hence may be but ten short years, will bring to life, and shed bright luster on the fortunate, who, reading in some volume, long since out of print, the secret hint and dull suggestion, forces by the principle of mind over matter, what is reaped by generations as a benefit of countless worth.

The use of water as an agent of titanic power or a mild persuasive, never-yielding laborer, is most instructive in its deeply philosophical deductions. For the windmill whose most mighty sweep and useful ends, which are uncertain as the variation of this curious method of time-saving theories, the water wheel is ever ready to respond and work on with even tenor neither more nor less than man dictates. The over-shot or under-shot prove useful as the case demands, or the locality suggests. That simple law of gravity continues to move mighty cupped circumferences with their multiplied smooth cogs, thus crushing iron; rolling out the tenuated wire, and, in some instances, turning out by easy efforts sixty horse shoes every minute.* When the little stream has passed the foundry and busy village; fed the workman's cattle and secured a competency for the rudest journeyman; it still moves on with playful ripple till the genial rays of sunshine purity call up its vapors to the fleecy clouds where it remains in dreamy listlessness till needed for descent upon some lofty region, when again it follows out the laws of nature and pursues its course with gentle murmurings.

In ancient times the aqueduct formed some of the most noted works of mighty Emperors. Cognizant of the vast power of water, when confined to lonesome duties, it became the business of those persons, who secured to city privileges the great blessing of a purest beverage, to protect surrounding neighborhoods from too great overflows of liquid strength.

The Aqua Marcia, in length some sixty miles, displayed the genius of a master mind† when Roman power breathed above subjected forces. Agrippa's baths derived much comfort from the celebrated Aqua Virgo; and the Aqua Claudia, about forty-six miles long, will be remembered by the student of antiquity with feelings of astonishment and emotion of a pleasurable character.‡

According to Prof. Anthon's lucid work,|| "when all the aqeducts were in operation, the quantity of water must have amounted to 50,000,000 cubic feet." The strictest supervision of such useful and most needful works must have proved no sinecure to the circuitores, tectores, castellarrii, villici and sillicarii, all subservient to the respected præfecti aquarum. These mighty agents of vitality were esteemed of more importance to the public welfare than the much loved pursuits of war, or the most fascinating arts of peace.

Surely, if the gifted Lamb could say, with truth, the city's parks are its vast lungs; with equal strength, in metaphorical allusion, might we call the High bridge, near New York, with its many pipes and flowing health, the inhabitants' thoracic duct, carrying nourishment to those of feeble constitution and life to the exhausted.

The toggle-joint, employed by workmen, is possessed of countless benefits, and by mysterious principles involving the

---

* Burden's Factory, Troy, N. Y.

† Frentin, de Aqueduct: Plin. H. N.—Dion. Cass: Vitruv., viii. 3, 1.

‡ Smith, Dict. ant.; Becker, Handb. d. Rom. Alterth.

|| Roman Antiquities—Aqueducts.

parallelogram of forces, brings about the happiest results; the steam-press is all powerful in its purposes—this vapor of water being most accommodating. The lever-press can perform the labor of many men, according to the position of the fulcrum and the length, etc.; but the acme of force, the resultant of exhaustive power, the product of a multiplied and never yielding influence, is only to be found in the hydraulic press. By it cannon are most surely tested, and commodities confined within the smallest space. As another proof of the sublime power of water, when an earthly being seeks to confine it to a mercantile existence, a reservoir but *one inch wide*, that is, with the capacity to hold a column of water one inch wide and some fifty feet high, would require as thick walls and demand as careful masonry as if the building were erected to contain a mile square of water with its countless millions of gallons, that is, if it be of the same depth. The simple example of the glass tube one hundred feet high, containing a column of water, and poured into a barrel already full, bursting it, though strongly made, at once explains the phenomenon.

Huge rocks have been torn asunder by the mere insertion of a few wedges left exposed to the weather. The first rain is absorbed by the pores of the wood, and, expanding, an incredible force is displayed, wonderfully energetic and successful as to practical results. Powder, no doubt, would do as much, but the gradual persuasion of the swelling properties of wood accomplish the desired end more surely, and with less risk of breaking off what was not deemed necessitous.

While molten lead contracts when cool, obedient to the laws of heat, water after reaching the depressing temperature of 28°, its smallest limit, expands accordingly, and when the freezing point has been obtained, just 32°, many a vessel, be it glass or iron, has been fractured by this immense power. The philosophical experiment of dropping oil into a pool of water, and its consequent spheroidal shape, produces much of import to the strict deducer of important fruits from little seeds of thought. The rotundity of shot and the utility of water in arresting its formation are of vast importance in a scientific way, to him who seeks to prove the shape of earth, the magic form of matter as created out of chaos.

Of all the methods employed for the purpose of determining the specific gravity of solids, not excepting the assistance obtained formerly from Nicholson's areometer, since the volumetric assay, and graduated cylinders of recent date, F.

Mohr, a man of genius and peculiar skill, has arrived at the conclusion that the simplest is to measure the volume of water displaced, whereas, before, it was the custom carefully to weigh.

As an additional proof of the power of water, in the shape of waves, masses of rock weighing nearly ten tons have been heaped up by the agency of storms, etc., some sixty-two feet above the sea.*

Professor Tyndell, whose imagination is only equalled by his beauty of thought, has expressed the following sentiment relative to the phenomena of waves:

"When the tide advances over a sea-beach on a calm and sunny day, and its tiny ripples enter at various points the clear, shallow pools which the preceding tide left behind, the little wavelets run and climb and cross each other, and thus form a lovely chasing, which has its counterpart in the lines of light converged by the ripples upon the sand underneath. * * * By the action of interference its surface is sometimes shivered into the most beautiful mosaic, shifting and trembling as if with a kind of visible music." Such suggestions to the poetic mind place man upon a footing far above the details of a sublunary life. By entertaining meditations of this character, we rise to that pure sphere where we meet, with grateful feelings, a responsive tone in the works of Tieck, and cherish thoughts that exist as rarely as true virtue!

The deep-sea thermometer† reveals most curious facts connected with the temperature of depths and effects of air and light on what is found above the surface of the watery world. In treating of the causes of cold on high mountains, M. Martins remarks that the radiation of mountains is much greater when covered with snow, particularly above where there exists any melting process.‡ Flocculent snow, however, does not seem to possess this property. With reference to the mechanical theory of heat, again, we find that, in connection with the relations of heat and work, the investigations of Mr. Joule, of Manchester, would be as nothing were the friction of water not included in his investigations. "Joule's Equivalent," for various thermometric scales, in English and French units, can not fail to be suggestive:

---

* Thomas Steveson's (C. E.) Communication to the Royal Society.

† The newest is that devised by W. Scott, of England.

‡ Anneles de Chimie et de Physique.

1 English thermal unit, or 1 deg. Fah. in 1 ℔ of water, .772 ft-pounds.
1 centigrade deg. in 1 ℔. of water, 1389.6 foot-pounds.
1 French thermal unit, or 1 centigrade degree in a kilogramme of water, 423.55 kilogramme-metres.

It is only by the accumulation of such data, and the thorough digestion of equally important statistics, that we learn how to adapt our frames to the endless variety of conflicting elements and conducive principles. Even as regards the separation of the heat and light of the sun's rays in the *eye*, J. M. Janssen,* as a result of his experiments, proves conclusively that *all* the rays of heat are absorbed before they reach the retina—two-thirds by the cornea, and the other third by the *aqueous* humor."

In the analysis of induction sparks, M. Moncel, of France, found, by careful investigations, that on plunging one of the "rheophores" into water, and if the discharge were taken through the fluid, the "luminous atmosphere exhibited singular corruscations."

A most remarkable theory has been maintained by M. Perrot, of Paris, relative to the earth's annual motion, which is far more interesting than the pendulum experiment of Mr. Foucault, or the more complicated labors of M. Fitzeau. A bucket is suspended in the air. A small hole in the exact center of its bottom is made; water is then poured in, and a minute quantity of powder strewn on the surface, for the purpose of detecting the course of the current. The careful originator of this interesting experiment ascertained that the current followed the curve very much to the right of a straight line, such as would be found to be the case were the earth standing still. Founding his premises upon these facts, M. Perrot asserts that the earth's rotation may be discovered by watching the course of rivers. Invariably the right bank is pressed upon.

To those enamored of the subject of capillary attraction, the views of Mr. Sorby, relative to the movements of fluids in porous bodies, suggest even electrical phenomena, almost paradoxical their character. It has been ascertained, in Germany, that ice one inch and a half in thickness, will bear the weight of a man; three inches and a half, a detachment of infantry; four and four-tenth inches will support the passage of eight pounders; five and two-tenth inches, twelve pound-

---

* Academie des Sciences.

ers; eight inches, twenty-four pounders, and twelve inches, almost any resistance from above.*

Science has made rapid strides of late respecting the laws of light, electricity, and heat, but the views of Dr. Scott Alison, before the Royal Society, "on the intensification of sound through solid bodies by the interposition of water between them and the distal extremities of hearing-tubes," afford surprising proofs of its value as regards assisting man in clearly demonstrating all the necessary waves of sound as found among the works of learned men concerning the acoustic principle. The phonoscope and hydrophone unfold a storehouse to inquiring students. The figures produced by sound, as acting upon water in a vessel, are of a certain size and shape, according to the intensity or pitch.†

Few facts exist that seem more subtle in their nature, more eccentric in their composition, than the many different opinions of variously learned men relative to the wave-line theory, that is, the question as to what "became of all the water which a ship removed out of her way?"‡ The following table concerning "the velocity with which the traveling wave moves, and which is found to depend entirely on the depth of the water," may prove of interest to nautical observers:

| | | | | | | | |
|---|---|---|---|---|---|---|---|
| At | 3 | feet deep | the wave | travels | 6 | miles | an hour. |
| " | 5 | " | " | " | 8 | " | " |
| " | 7 | " | " | " | 10 | " | " |
| " | 10 | " | " | " | 12 | " | " |
| " | 15 | " | " | " | 15 | " | " |
| " | 20 | " | " | " | 18 | " | " |
| " | 30 | " | " | " | 20 | " | " |
| " | 40 | " | " | " | 25 | " | " |
| " | 50 | " | " | " | 30 | " | " |

INDIA-RUBBER STOPPERS FOR BOTTLES. *Messrs. Editors*: Will you allow me to call the attention of the profession, through your pages, to a recent invention, which promises to be of great convenience to physicians, as well as to all other classes of the community. I refer to the India-rubber corks for bottles and vials. By means of this admirable substitute for the common cork, bottles containing the strongest acids

* Annual of Scientific Discovery, 1861. † Poggendorff's Annalen.
‡ See J. Scott Russell's Communication before the British Institution of Naval Architecture.

